# Direct gastric invasion from the liver metastasis of colorectal origin: A case report

**DOI:** 10.1097/MD.0000000000037732

**Published:** 2024-04-12

**Authors:** Jun Yeb Nam, Jung Wook Lee, Jae Hyun Kim, Minjung Jung, Moo In Park, Won Moon, Sung Eun Kim, Kyoungwon Jung, Seun Ja Park

**Affiliations:** a Department of Internal Medicine, Kosin University College of Medicine, Busan, Korea; b Department of Pathology, Kosin University College of Medicine, Busan, South Korea

**Keywords:** colorectal cancer, conversion therapy, direct gastric invasion, liver metastasis, Repeat hepatectomy

## Abstract

**Rationale:**

Colorectal cancer is the third most common cancer diagnosed worldwide. At the time of diagnosis of colorectal cancer, one of the most common metastatic sites is liver. Gastric metastasis from colorectal origin is rare. Moreover, a direct invasion of the stomach, by hepatic metastasis from colorectal cancer, is particularly uncommon.

**Patient concerns:**

A 56-year-old male patient with hematochezia was referred to our hospital.

**Diagnosis:**

The patient was diagnosed with unresectable colorectal cancer because of the presence of >10 metastases involving both lobes of the liver.

**Interventions and Outcomes:**

After chemotherapy, the metastatic nodules in the liver nearly disappeared, except for a small nodule in segment VI. The patient underwent a radiofrequency ablation for the single lesion in the liver and laparoscopic low-anterior-resection for the primary tumor. Despite receiving various chemotherapy regimens, he experienced 6 recurrences, leading to 5 hepatectomies including a right hemi-hepatectomy, 1 pulmonary wedge resection, and 2 courses of radiation treatments. Lastly, a metastatic lesion in the liver was observed with invasion into the stomach. Subsequently, gastric wedge resection with resection of segments III and IV of the liver was performed. Direct invasion of the liver metastases into the stomach was confirmed histologically.

**Lessons:**

The patient is still alive, with a good quality of life, even after more than 8 years since the initial diagnosis. In the last instance of metastatic recurrence, direct invasion from the liver metastases into the stomach was observed, which is rare, and there are currently no reported cases.

## 1. Introduction

Colorectal cancer is the third most common cancer diagnosed and ranks second in terms of incidence worldwide.^[1]^ In South Korea, colorectal cancer has the second highest cancer incidence rate and the 4th-highest cancer mortality rate.^[2]^ At the time of diagnosis, approximately 20% of patients have distant metastatic disease, and the most common metastatic sites are regional lymph nodes, liver, lungs, and peritoneum.^[3]^ According to Korean statistics (2016–2020), colorectal cancer with distant metastases has a 5-year relative survival rate of 20.4%.^[2]^ If both the primary tumor and liver or lung metastases are resectable, National Comprehensive Cancer Network guidelines suggest surgical resection as the curative treatment.^[4]^ Apart from surgical resection, local therapies considered include stereotactic body radiotherapy (SBRT) and radiofrequency ablation (RFA).^[5]^ When metastatic lesions are unresectable, systemic chemotherapy is recommended. Testing for RAS and BRAF mutations is essential because these results can help guide the selection of a chemotherapy regimen.^[6,7]^ Conversion therapy is considered when systemic chemotherapy has a good response for initially unresectable colorectal cancer. Conversion therapy was defined as macroscopically complete removal of all lesions disseminated from the colorectal cancer and the primary tumor that had initially been deemed unresectable.^[8]^ Gastric metastasis from colorectal origin is rare.^[9]^ Direct invasion of the stomach by hepatic metastasis from colorectal cancer is particularly uncommon.

Herein, we report a case that underwent conversion therapy, repeated hepatectomies (5 times, including segmentectomy), video-assisted thoracoscopic surgery (VATS) of both lungs, SBRT, and had 6 types of chemotherapy regimens for approximately 8 years. In the last instance of recurrence, we observed a direct invasion of the stomach from hepatic metastatic site, for which stomach wedge and liver resections were performed.

## 2. Case report

A 56-year-old male patient with hematochezia was referred from a local clinic and was admitted to our hospital in May 2015. The serum carcinoembryonic antigen (CEA) was slightly elevated (6.12 ng/mL). Colonoscopy revealed a circumscribed mass approximately 40 × 30 mm in size with sharp margins and central ulceration in the rectum, about 15 cm from the anal verge. Histological examination of the biopsy specimen revealed well-differentiated adenocarcinoma. No KRAS mutation or epidermal growth factor receptor mutations were detected. The patient was diagnosed with unresectable colorectal cancer because of the presence of >10 metastases involving both lobes of the liver. Palliative treatment with the bevacizumab-FOLFIRI(irinotecan, levofolinic acid, and 5-fluorouracil) chemotherapy regimen was chosen. In the response evaluation after the 13th chemotherapy session (November 2015), the outcome demonstrated significant improvement, with the metastatic nodules almost disappearing, except for a small 1 cm nodule in the segment VI of liver. Therefore, we then switched to conversion therapy as the curative approach. RFA of the single liver lesion was performed in November 2015, and resection of the primary colon tumor was planned. On completing 15 cycles of chemotherapy, laparoscopic low-anterior resection was performed (January 2016). Pathological examination confirmed well-differentiated adenocarcinoma. None of the 13 resected regional lymph nodes showed any signs of metastasis. The pathological tumor node metastasis stage according to the Union for International Cancer Control was ypT3N0M1a. Microsatellite Instability was assessed and was determined to be stable. The patient refused to undergo adjuvant chemotherapy.

**Figure 1.**
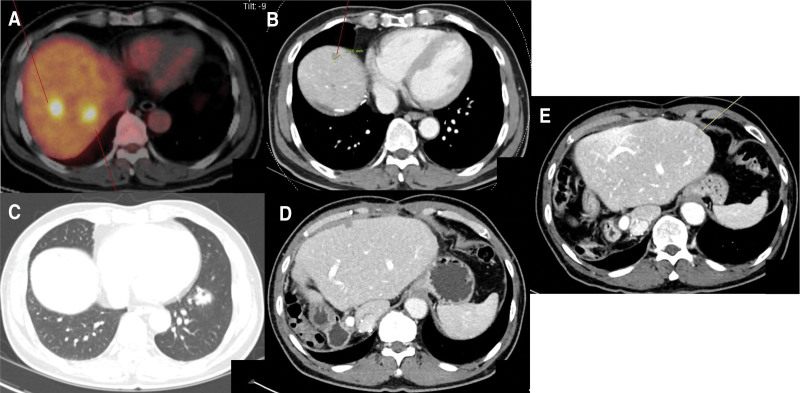
PET-CT or abdominal CT images. (A) Multiple metastases in the right lobe of liver in the PET-CT. (B) A metastatic nodule in the residual liver in the CT. (C) Metastatic nodules in both lower lobes in the CT. (D) A metastatic nodule in the residual liver (anterior subcapsular area) in the CT. (E) Hepatic metastasis in the left liver (subcapsular area) in the CT. CT = computed tomography, PET = positron emission tomography.

After surgery, the patient was evaluated every 3 months using abdomen and chest computed tomography (CT) and serum CEA levels. In September 2016, abdominal CT and positron emission tomography (PET)-CT revealed multiple metastases to the right lobe of the liver (Fig. 1A). The patient’s CEA was 9.53 ng/mL. As multiple liver metastases were confined to the right lobe, a right hemi-hepatectomy was performed in September 2016. Histological examination revealed metastatic adenocarcinoma. Following surgery, the patient received 12 cycles of bevacizumab-FOLFOX4 (fluorouracil, folinic acid, and oxaliplatin) chemotherapy regimen contained between October 2016 and May 2017.

A second metastatic recurrence was detected in the abdominal CT in August 2017, with approximately 1 cm-sized newly developing nodule in in segment II of the residual liver (Fig. 1B). The patient underwent a partial hepatectomy in August 2017 followed by third-line chemotherapy using single-agent capecitabine from September 2017 to March 2018 (9 cycles).

A CT scan in July 2018 revealed metastatic nodules in both lower lobes of the lungs (3) and in segment III of the residual liver (anterior subcapsular area) (Fig. 1, C and D). Surgery was planned for both lower lung lesions and SBRT for the liver lesion. VATS of the right lower lobe, wedge resection, and left lower lobe anteromedial segmentectomy were performed. SBRT was administered in August 2018 (6000 cGy in 4 fractions of 1500 cGy each). Subsequently, the patient received 12 cycles of cetuximab-irinotecan chemotherapy regimen as the fourth line from September 2018 to March 2019.

The patient’s performance was good after the 4th chemotherapy until April 2020, without recurrence. In April 2020, a new hepatic metastasis was detected in the subcapsular area of the left liver (Fig. 1E). A laparoscopic wedge resection of the liver was performed in May 2020. Histological examination showed cancer involvement in the resection margins. Although further chemotherapy or radiotherapy was considered, but it was not feasible.

**Figure 2.**
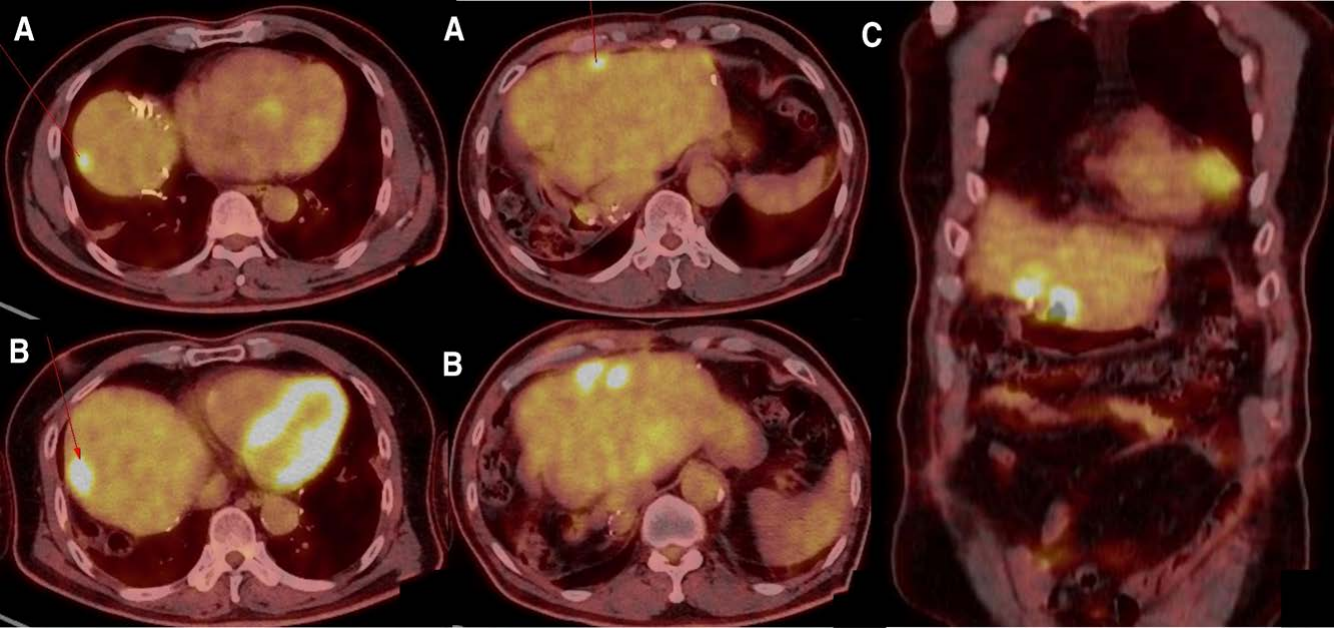
PET-CT images. (A) Focal hypermetabolic lesions in the remnant liver. (B) Three hypermetabolic lesions in the liver (axial view). (C) Metastatic nodules close to stomach (coronal view). CT = computed tomography, PET = positron emission tomography.

**Figure 3.**
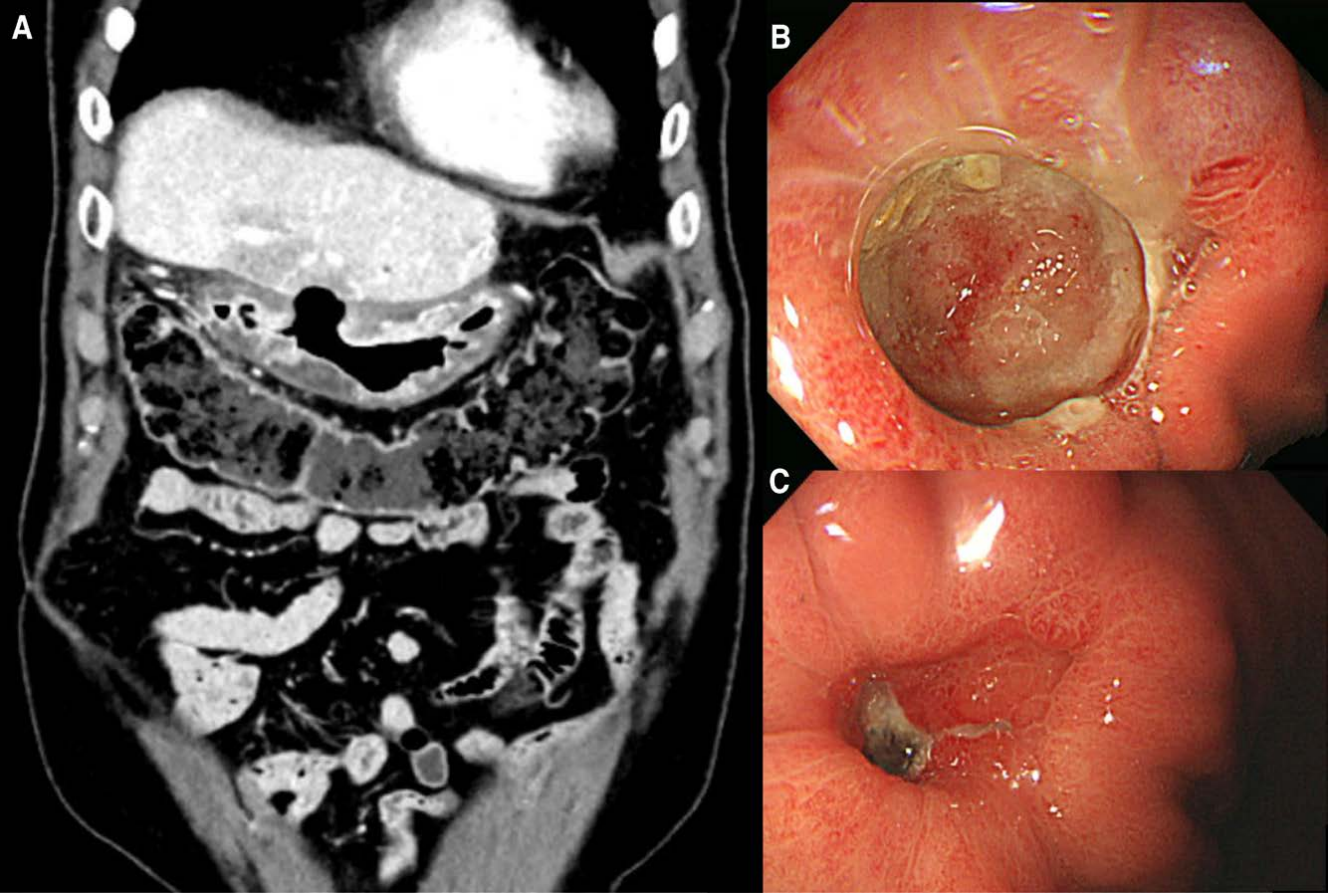
CT and gastroendoscopy image. (A) A deep ulcer in the stomach in the CT (coronal view). (B) A deep ulcerative lesion in the gastroendoscopy. (C) Follow-up gastroenteroscopy image. CT = computed tomography.

The 5th metastatic recurrence was detected in January 2021. Newly developed focal hypermetabolic lesions in the remnant liver were observed on PET-CT (Fig. 2A). In view of the several previous surgeries and the presence of multiple lesions in unfavorable locations, the surgical team concluded that a clean excision would be impossible. Hence concurrent chemoradiotherapy was offered with a total dose of 6500 cGy in 26 fractions of 250 cGy each, from March 2021 to April 2021. Two cycles of FL (5-fluorouracil and leucovorin) chemotherapy were administered concurrently. A CT scan on June 8, 2021, after concurrent chemoradiotherapy showed that the previously observed metastatic nodule had decreased in size, but a deep ulcer in the stomach was detected (Fig. 3A). Gastroendoscopy revealed an approximately 25 × 20 mm-sized round-shaped deep ulcerative lesion with fold convergence at the anterior wall and greater curvature of the lower body (Fig. 3B). Although the lesion was highly suspicious of malignancy, biopsy results indicated ulcer debris. On follow-up endoscopy (3 months later), the ulcer base appeared to have improved, and was approximately half filled with regenerative epithelium(Fig. 3C). Histological examination was consistent with that of ulcer scars. On the basis of these results, we concluded that the lesion was benign.

There were differences in the findings among the imaging modalities of the liver (CT, magnetic resonance imaging [MRI], and PET-CT) after the 5th metastatic recurrence. Table 1 shows the readings by the radiologists based on when and how they were taken. In July 2021, all 3 modalities confirmed the absence of metastatic recurrence. In April 2022, a PET-CT showed a new focal hypermetabolic lesion in the liver and CT showed a rim enhancing nodule in the same lesion. The radiologist opined that the lesions observed were most likely inflammatory changes. In October 2022, the postradiotherapy site was observed to be reduced in size on liver MRI, and there was no significant difference from the previous scan on liver CT. However, on PET-CT, the existing hypermetabolic lesion was enlarged and a new lesion was observed in the anterior subcapsular area. After the multidisciplinary care meeting, it was concluded that additional surgeries and further radiation therapy would be unreasonable because the patient had already undergone multiple surgeries and radiation treatments. In January 2023, on PET-CT, the previously observed hypermetabolic lesions were more prominent and new lesions were observed; while MRI and CT of the liver did not show any significant differences from previous studies. However, in April 2023, liver MRI showed enlargement of the enhancing lesion, and PET-CT, a further increase in the metabolism of the lesion, finally confirming a metastatic recurrence rather than an inflammatory change.

**Table 1:** Formal readings and findings based according to time and modality.

	2021.07.	2022.04.	2022.10.	2023.01.	2023.04.
Abdomen CT					
Readings	Radiotherapy change	Tumor recurrence or inflammatory change	Tumor recurrence or inflammatory change	Tumor recurrence or inflammatory change	Tumor recurrence or inflammatory change
Findings	Decrease size of nodules at the liver	Rim enhancing nodule in the remnant liver	Equivocal change of rim enhancing nodule	No interval change of rim enhancing nodule	No interval change of rim enhancing nodule
Liver MRI					
Readings	Probable radiotherapy change	No interval change of radiotherapy change	Interval decrease in size of radiotherapy change	Tumor recurrence or inflammatory change	Viable tumor or radiation induced change
Findings	Radiotherapy change in the liver	Radiotherapy change in the liver	Radiotherapy change in the liver	No interval change of peripheral enhancing lesions at the liver, radiotherapy site	Increased size of peripheral enhancing lesions at the liver, radiotherapy site
PET-CT					
Readings	Improved state	Liver metastasis, suggestive	Liver metastasis, suggestive	Liver metastasis, suggestive	Liver metastasis
Findings	Disappearance of hypermetabolic liver metastases	Focal hypermetabolic lesion reappeared	New focal hypermetabolic lesion in the anterior subcapsular area of the liver (total 2)	More prominent hypermetabolism of hepatic metastases, newly developed hepatic lesion (total 3)	Increased metabolism compared with the previous study (2023.01.)

CT = computed tomography, MRI = magnetic resonance imaging, PET = positron emission tomography.

**Figure 4.**
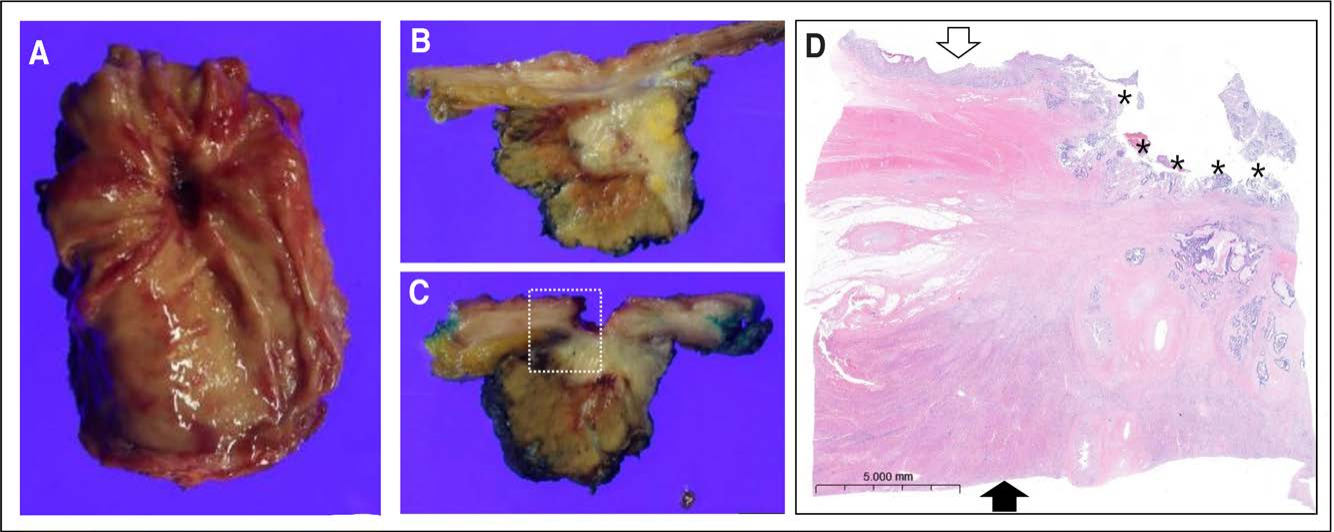
Pathologic findings of wedge resection of stomach and liver. (A) A deep, round ulcer is found in the gastric mucosa. (B) An ill-defined, solid mass is found in the hepatic tissue. (C) The hepatic mass directly invades to the adjacent gastric wall, forming a distinct ulcer. (D) Microscopic finding of the rectangular dotted area in C reveals adenocarcinoma spanning the hepatic parenchyma (black arrow) and gastric wall (white arrow). Asterisk (*) indicates the gastric ulcer produced by adenocarcinoma. (Hematoxylin and eosin stain, X6.).

Three hypermetabolic lesions were observed in the liver. One of these was located close to the stomach (Fig. 2B and C). The level of serum CEA was elevated at 16.90 ng/mL. Bevacizumab–Lonsurf chemotherapy regimen, as 6th line, was started in May 2023. Meanwhile, follow-up gastroendoscopy, performed in August 2023, showed worsening of the previously observed ulcer. A forceps biopsy indicated a well-differentiated adenocarcinoma. A multidisciplinary meeting attended by physicians from the departments of internal medicine, surgery, radiation oncology, and nuclear medicine discussed the lesions in the liver and the stomach and concluded that the liver lesions were metastases of colon cancer. Further, the lesions in the stomach were deemed to be a direct invasion from the liver into the stomach. A wedge resection for liver metastases and the directly invaded lesions in the stomach was agreed to be the best option to preserve gastric function in the meeting. Following surgery, radiotherapy was recommended for the remaining lesions. Gastric wedge resection with resection of segments III and IV of the liver was performed in October 2023. Two ill-defined, solid masses, measuring 2.2 × 1.6 and 3.2 × 3.2 cm, were found in segments III and IV of the resected liver, respectively. Microscopically, they were adenocarcinomas immunoreactive for MOC31, CK20, and CDX2, and their final pathologic diagnosis was adenocarcinoma of colorectal origin. In the case of adenocarcinoma of the segment III, the epicenter was in the hepatic tissue, however, it extended directly into the adherent stomach, forming an ulcer (Fig. 4). The patient was discharged 2 weeks after surgery and scheduled for outpatient follow-up. The patient is still alive, over eight years after the initial diagnosis, with a good quality of life.

## 3. Discussion

**Figure 5.**
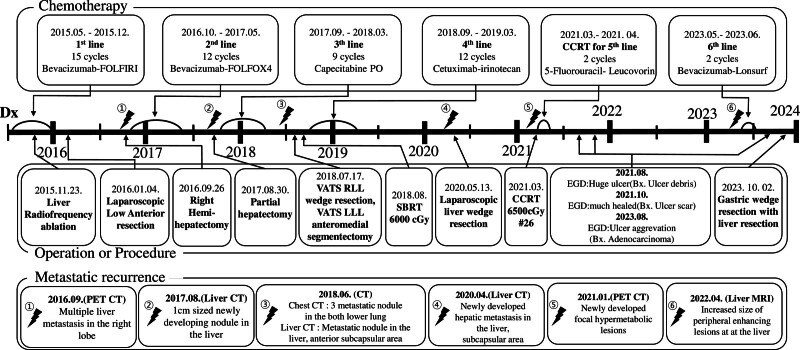
Events according to the 3 categories (chemotherapy, operation or procedure, metastatic recurrence) in chronological order in horizontal axis. A lightning bolt symbol was marked on the horizontal axis when metastatic recurrence occurred. In “chemotherapy” part, we displayed the type of regimen, duration, and cycles of chemotherapy. The “Operation or procedure” part contains the name of the surgery or procedure and date. In the “Metastatic recurrence” part, we indicate the date of the new lesion and the modality performed. Bx  = biopsy result, CCRT = concurrent chemoradiotherapy, CT = computed tomography, Dx = diagnosis, EGD = esophagogastroduodenoscopy, FOLFIRI = irinotecan, levofolinic acid, and 5-fluorouracil, FOLFOX4 = fluorouracil, folinic acid, and oxaliplatin, LLL = left lower lobe, MRI = magnetic resonance imaging, PET = positron emission tomography, RLL = right lower lobe, SBRT = stereotactic body radiotherapy, VATS = video-assisted thoracic surgery.

The patient experienced 6 recurrences of metastasis and underwent multiple surgeries (colon surgery, VATS, and 5 liver resection), 1 RFA, and 2 courses radiotherapy. Six different chemotherapy regimens were administered. Figure 5 depicts the entire course of treatment as a flowchart with the time scale, providing a glance at the treatment history.

Testing for RAS and BRAF mutations at the time of colorectal cancer diagnosis is crucial because it helps to determine the appropriate chemotherapy regimen. For example, patients with metastatic colorectal cancer, who are wild-type for both RAS and BRAF genes, are the best-suited candidates for anti-epidermal growth factor receptor treatment.^[6,7]^ Initial therapy with cetuximab-based regimens showed benefits for RAS wild-type left-sided tumors in a meta-analysis of 3 randomized trials that directly compared chemotherapy with either cetuximab or bevacizumab.^[10]^ Our patient received bevacizumab despite having RAS wild-type, left-sided tumor due to limitations in the public healthcare system in South Korea. Regimens not covered by public health insurance require the patient to pay the full expense leading to a financial burden. Cetuximab was not covered by insurance at the time of diagnosis; therefore, it was unavailable.

At the time of diagnosis, the colorectal cancer was deemed unresectable because of the presence of >10 metastases involving both liver lobes. The positive response of the patient to the chemotherapy, with only 1 remaining liver lesion besides the primary tumor upon completing the regimen, enabled us to proceed with surgery. A retrospective study evaluated 99 patients with unresectable metastatic colorectal cancer who received first-line chemotherapy. Twenty-three (23.2%) patients were able to undergo conversion surgery. The presence of liver metastases, single-organ metastases, and the use of biological agents such as bevacizumab and cetuximab were independent predictors of successful conversion therapy. The long-term survival among patients who underwent successful conversion surgery was comparable to that of patients who had surgery as the initial treatment for resectable stage IV colorectal cancer.^[8]^ Furthermore, in our case, the patient met all 3 of the above conversion success factors.

Our patient underwent repeated hepatectomies (5 times). Wicherts et al^[11]^ studied the oncological benefits of repeated hepatectomies in patients with recurrent colorectal metastases. A total of 645 patients had undergone a single hepatectomy, while 288 patients had repeated hepatectomies (225 had 2 hepatectomies, 52 had 3 hepatectomies, and 11 had 4 hepatectomies). The overall survival rates at 3 and 5 years after the first hepatectomy were 76% and 54%, respectively, for patients who underwent repeat hepatectomies, in contrast to 58% and 45%, respectively, for those who had a single hepatectomy (*P* = .003).^[11]^

Confirmation of recurrence was delayed owing to disparities among imaging modalities. In summary, it can be concluded that the recurrence occurred in April 2022 when PET-CT showed abnormalities. Elevation of serum CEA (5.25 ng/mL) further supported the presence of recurrence. We had to be careful because the patient had just received radiation in the same area, and required time to recover. Multiple surgeries, chemotherapy, and radiation caused confusion in the interpretation of the imaging findings. Brook et al^[12]^ studied the radiological response to stereotactic radiotherapy in focal liver tumors, including metastatic lesions. The liver parenchyma adjacent to the lesion demonstrated a prominent halo of delayed enhancement in 19/21 (91%) of metastases.^[12]^

The metastasis to the stomach is rare, with a reported incidence of only 1.7% in autopsy cases.^[9]^ The metastatic spread of original primary cancers to the stomach involves 4 pathways: peritoneal dissemination, hematogenous dissemination, lymphatic spread, and direct tumor invasion.^[13]^ Yoshimi et al^[14]^ presented a case report, very similar to our case, of long-term survival after repeat resections of metastases in the liver, lung, and stomach from sigmoid colon cancer. The patient underwent colectomy for sigmoid colon cancer, repeated hepatectomies (2 times), and pulmonary resection for the metastases. Gastric metastasis was detected, and partial gastrectomy was performed. Gastric metastasis was concluded to be through hematogenous spread, given its primary location in the proper muscle layer of the stomach, minimal lymphatic permeation, and the absence of other intraperitoneal lesions. To our knowledge, our case is the only reported instance in which liver metastases from colorectal cancer have spread directly to the stomach.

Several cancer of direct invasion to stomach have been reported previously. Terashima et al^[15]^ reported a case of gastric metastasis originating from transverse colon cancer. In the case reported by Kimura et al,^[16]^ a patient diagnosed with hepatocellular carcinoma was hospitalized for abdominal pain and underwent an upper endoscopy, which revealed a massive protruding lesion. Abdominal CT showed an extrahepatic enlarged mass with invasion into the pancreas and stomach.^[16]^ The major factors in direct invasion included the growth pattern, size, and location of the masses. In most cases of direct invasion, the masses are large, located in the subcapsular area, and exhibit an exophytic pattern.^[17]^ In the present case, the liver metastasis was located in the capsular area, although the size was not large.

Kim et al^[18]^ studied the endoscopic features of metastatic tumors in the stomach. The leading cause of metastatic tumors in the stomach was malignant melanoma, accounting for 10 cases (27.0%), followed by lung cancer with (7 cases, 18.9%) and breast cancer (5 cases, 13.5%). The endoscopic appearance of metastatic lesions was divided into 2 main categories. One resembled submucosal tumors (12 cases, 32.5%) and the other (25 cases, 67.5%) resembled primary gastric cancer.^[18]^ In our case, the lesion appeared to be a deep ulcer.

Our patient was deemed inoperable at the initial diagnosis; however, after chemotherapy, conversion surgery was possible. Since then, he has experienced several recurrences of metastases, but has had a long survival with multiple surgeries and chemotherapy. The patient is still alive, with a good quality of life, even after more than 8 years since the initial diagnosis. In the last instance of metastatic recurrence, direct invasion from the liver metastases into the stomach was observed, which is rare, and there are currently no reported cases.

## Author contributions

**Writing—original draft:** Jun Yeb Nam, Jung Wook Lee.

**Writing—review & editing:** Jae Hyun Kim, Sung Eun Kim, Kyoungwon Jung, Seun Ja Park.

**Visualization:** Minjung Jung.

**Supervision:** Moo In Park, Seun Ja Park.

**Conceptualization:** Won Moon, Seun Ja Park.
